# Supplementary material for: Identification of photoperiod-induced specific miRNAs in the adrenal glands of Sunite sheep (Ovis aries)
Source: Front Vet Sci. 2022 Jul 22;9:888207. doi: 10.3389/fvets.2022.888207 (PMC9354845; doi:10.3389/fvets.2022.888207)
Supplement: Supplementary file 1 [file Data_Sheet_1.ZIP › Supplementary file/miRDeep2 file.zip!/miRDeep2 file/Novel_Structure/SP42A2/Novel_71.pdf]

Provisional ID	:	NC_019463.2_16740
Score total	:	2
Score for star read(s)	:	-1.3
Score for read counts	:	0
Score for mfe	:	1.7
Score for randfold	:	1.6
Score for cons. seed	:	
Total read count	:	33
Mature read count	:	30
Loop read count	:	0
Star read count	:	3

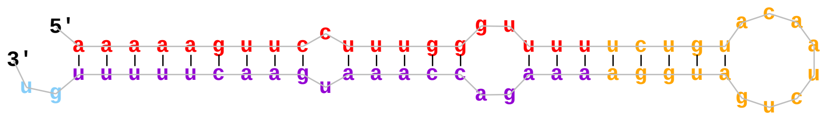

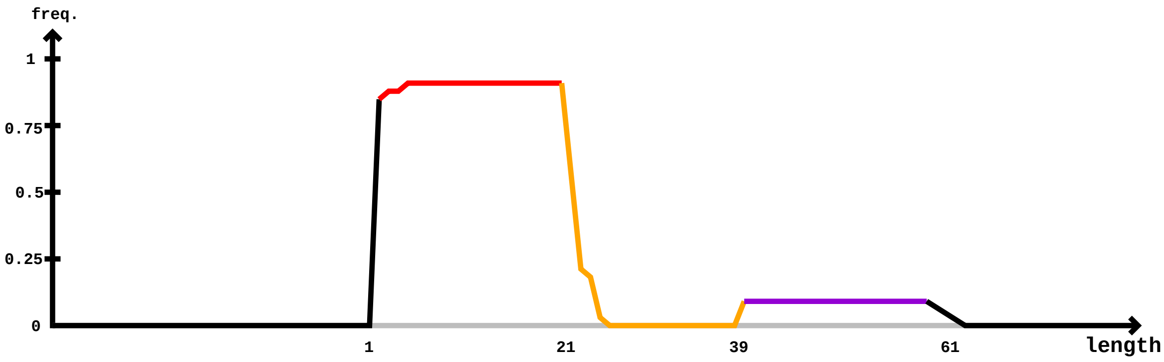


```
                                   Mature                                Star
5'- aauaucuauuucaaguaucuauuggguuggacaaaaaguuccuuuggguuuuucuguacaaucugauggaaaagaccaaaugaacuuuuuguuaaauccaauauuuauuca   -3'    obs
    aauaucuauuucaaguaucuauuggguuggacaaaaaguuccuuuggguuuuucuguacaaucugauggaaaagaccaaaugaacuuuuuguuaaauccaauauuuauuca          exp
    .............((((..(((((((((.((((((((((((.(((((..((((((((........))))))))..))))).)))))))))))).)))))))))..))))..    reads      mm       sample
    ................................aaaaaguucGuuuggguuuu...........................................................    23         1        seq
    ................................aaaaaguucGuuuggguuuuucu........................................................    5          1        seq
    .................................aaaaguucAuuuggguuuuuc.........................................................    1          1        seq
    ...................................aaguucGuuuggguuuuucug.......................................................    1          1        seq
    ......................................................................aaagCccaaaugaacuuuuu.....................    2          1        seq
    ......................................................................aaagaccaaaugaacuuuuug....................    1          0        seq
```
